# Youth Networks' Advances Toward the Sustainable Development Goals During the COVID-19 Pandemic

**DOI:** 10.3389/fsoc.2020.589539

**Published:** 2020-10-29

**Authors:** Kevin Barber, Mohammed A. Mostajo-Radji

**Affiliations:** ^1^The Rockefeller University, New York, NY, United States; ^2^Embassy of Science, Technology and Innovation, Ministry of Foreign Affairs of Bolivia, La Paz, Bolivia; ^3^Permanent Mission of Bolivia to the United Nations, New York, NY, United States; ^4^Clubes de Ciencia Bolivia Foundation, Santa Cruz de la Sierra, Bolivia

**Keywords:** science diplomacy, United Nations, non-state actors (NSAs), sustainable development goals—SDGs, youth networks, COVID-19

## Introduction

The emergence of the COVID-19 pandemic, a disease caused by novel coronavirus SARS-CoV-2 (Zhu et al., 2020), has presented major challenges to international public health systems. As new data surrounding the epidemiology of the disease continues to emerge, policy recommendations made by institutions such as the World Health Organization have been continually modified and inconsistently applied by national and local governments. For instance, in an effort to contain the initial spread of the disease internationally and reduce the rate of transmission domestically, early affected countries implemented policies to restrict travel and limit the social contact of their citizens (Acosta and Nestore, 2020; Anderson et al., 2020). While these strategies have been found to be effective at reducing the rates of new cases and minimizing the resulting strain to national healthcare systems (Chinazzi et al., 2020), global spread of the disease proved to be unavoidable. Yet specific responses enacted by national governments to mitigate the impact the pandemic would have on their society have varied (Anderson et al., 2020), and in cases, seemingly come in contradiction to one another. Inherent to these discrepancies appears to be a concern between limiting the health and mortality repercussions of the pandemic, opposed with reducing the economic impact associated with commercial and social restraint (Sheridan et al., 2020). The fractured international approach has highlighted the limitations established forms of governance have in agreeing upon and implementing legitimate measures of resolution during a rapidly evolving global pandemic, and how these limitations culminate in serious strains to international public relations.

Recently, the concept of transnational youth networks (TYNs) has emerged as a flexible solution to many of the obstacles faced by international and domestic governance (Acosta et al., 2020). Defined broadly as a cross-disciplinary professional group with members under the age of 40 and selected based on a competitive process that analyzes professional achievements, the hallmarks of TYNs maintain a versatile set of descriptions. These include the selection of members based on the merit of professional achievements, empowerment of decision-making capabilities toward achieving a defined goal, and the application of unique experiential knowledge of individual members (Acosta et al., 2020). Prior to the emergence of the COVID-19 pandemic, organization of TYNs was proposed as a means of driving forward public diplomacy, particularly in relation to the United Nations Sustainable Development Goals (SDG) agenda (Acosta et al., 2020). At the present moment, the preeminent objectives of the SDG agenda have been put in jeopardy by the unfolding pandemic (Naidoo and Fisher, 2020). Most relevantly, the objective of ensuring the health and well-being of global citizens through efficient funding and access to healthcare systems, along with increased sanitation and hygiene (Filho et al., 2020; Sustainable Development Goals, 2020). While the WHO and its affiliated partners have been paramount in providing recommendations to national governments' response to the pandemic, groups tantamount to TYNs have ascended to resolve many of the deficiencies left by broader governing bodies. Here we will provide an overview of exemplary solutions designed by these groups and present support for the continued backing of these types of networks as a mechanism to engage international crises. Though these groups do not fall definitively within our working definition of TYNs, we will argue that they contribute useful models of youth empowerment, nonetheless.

## Youth Networks and the COVID-19 Pandemic

Underlying the initial decision made by many national governments to enact extensive quarantining measures on citizens was a concern of overwhelming health care systems with growing rates of COVID-19 patient numbers. Accompanying predictions of shortages of health care system resources was an anticipation of a lack of available personal protective equipment (PPE) for health care workers. In the United States, while health care systems struggle to correspond with the national government for these supplies, a decentralized source of supply chain management was constructed by a coalition of young volunteers. To date, the C19 Coalition reports to have successfully supplied over 100 million PPE units to health care systems across the US and has formed a working collaboration with the National Governors Association, connecting governmental bodies with public and private organizations (Levy, 2020a). Additionally, creative solutions to develop new forms of PPE have been organized under the banner of #HackThePandemic and unified by hackthepandemic.org (Mensley, 2020). This organization allows volunteers and researchers across the globe access to 3D printable mask designs, as well as the ability to contribute large scale manufacturing capabilities of supplies and computing power aimed at the pursuit of understanding the disease through simulations of protein folding. The capability to 3D print effective mask designs has been embraced by high school and university students across the United States, including those in initially hard-hit cities such as New York and Seattle (Reid, 2020; Schlosser, 2020; Siegal, 2020). These techniques have been transferred and reproduced in other countries, particularly in the developing world (Tendencias El Tiempo, 2020). These initiatives provide outstanding examples of how the youth can adapt new technologies to contribute to solving dilemmas currently facing healthcare systems.

Along with shortages of PPE, a looming concern for international healthcare systems remains a lack of available ventilators, an essential piece of equipment for the treatment of critically ill patients (Truog et al., 2020). These inventory shortages have placed major strains on international diplomacy, as countries have exercised restrictions of the transport of ventilators across borders, and in some cases gone as far as resorting to the confiscation of other countries' ventilators during international transit (Kamdar, 2020). The developing world remains most vulnerable to these shortages (Breevoort et al., 2020), inspiring innovative design solutions for low cost and readily transportable ventilators from youth teams. These include ventures by US university students (Levy, 2020b; Rusch, 2020), as well as a team of young engineers from India, a country with an especially sparse ventilator inventory compared to the domestic population (Biswas, 2020). Additionally, a large review of these types of open source designs has been compiled for future adaption (Pearce, 2020). One of the most comprehensive networks involved in broad scale coordination between globally distributed volunteer groups working to design and produce PPE and medical supplies has been the Open Source Medical Supplies. This organization reports a current record of statistics related to the above venture, including the number of countries actively involved in local response efforts and the total amount of supplies that have been successfully delivered by the global community (Open Source Medical Supplies). Continuing to increase the supply and universal accessibility of ventilators will not only aid the treatment capabilities of underserved health care systems but may also begin to alleviate international diplomacy tensions that result from global medical equipment shortages. Of particular interest, is the focus youth networks have made on advocating for the attention to how regional aspects of the developing world pose specific challenges to coping with a spreading pandemic. For instance, populations living in high altitude regions such as Ethiopia, Ecuador, Bolivia, and Tibet, are sensitive to environmental conditions and physiological adaptations that influence the effectiveness of common ventilator design (Breevoort et al., 2020). Given the limited influence these regions have in the development of traditional health care equipment, special considerations will be necessary to design and supply ventilators capable of functioning effectively for high altitude populations.

Perhaps most at risk of suffering from a lack of specialized intervention in regards to the COVID-19 pandemic are refugee populations and people living in war zones (Jahanshahi et al., 2020). Given the poverty of health care related infrastructure, coupled with increased rates of underlying medical conditions and high population densities often found in refugee camps, the risk of infection within these zones has been a notably difficult problem for traditional governmental systems to address. To help facilitate screening capabilities of health care practitioners serving refugee populations, students at Harvard University have developed a software solution for the efficient collection and sharing of patient data (Parks, 2020). This technology has now become widely available and adapted to the needs of health practitioners within refugee camps across the globe. This development can be viewed as a pivotal first step to mitigating the severity of the pandemic on displaced populations, but more measures will clearly be needed to resolve the special vulnerability of these groups.

## Advancing the Sustainable Development Goals Agenda Through Youth Networks

In 2015, the United Nations put forward the SDG agenda as a blueprint to achieve a better and more sustainable future for all by 2030 (Joshi et al., 2015). While received with enthusiasm by most member States, the COVID-19 pandemic can potentially delay and even jeopardize the implementation of many of those goals (Naidoo and Fisher, 2020). Indeed, several analyses have revealed that the pandemic has worsened inequalities throughout the world and active interventions from governments are needed to address these issues (Ashford et al., 2020). To assist these needs, UNICEF has been at the forefront of organizing remote networks of youth volunteers inspired to rise to challenges specific to their communities (Hawke, 2020). The formation of this network, headed entirely by youth volunteers, was able to quickly progress through the stages of volunteer recruitment, identification of specific challenges, and response of clear-cut actions. This included initiatives that are aimed at safeguarding the mental health of the youth through access to online psychologists and creation of seed banks to be distributed for home gardening projects.

In the context of the COVID-19 pandemic, youth networks have primarily focused on SDGs 3, 4, 9, 10, and 17. Namely: Good health and well-being; quality education; industry, innovation and infrastructure; reduced inequalities; and partnerships for the goals. Yet, it is important to notice that SDGs are indeed interlinked and the development of one goal strengths the development of others (Dawes, 2020). Dynamic mathematical models have indeed shown that efforts to address goals 4–16 have a direct impact on goal 1, elimination of poverty (Dawes, 2020). As such providing access to healthcare technology has an impact on economic projections (Mahler et al., 2020). Using the examples pinpointed in this manuscript, one can see that 3D printing of PPE and ventilators, have not only addressed SDG 3, but inspired other regions in the world to adopt these technologies (addressing SDGs 4 and 9), consequently reducing inequality (SDG 10) and poverty (SDG 1).

## Obstacles Faced by Youth Networks in Gaining Governmental Support

Due to the characteristic of being non-state actors, youth networks are often left behind in policy and decision making (Carosso et al., 2019a). Indeed, one of the most common negative perceptions of young entrepreneurs and technology activists is the lack of governmental interest and support for their initiatives (Saucedo-Bendek et al., 2020).

The COVID-19 pandemic has further highlighted some of these issues. For instance, there is an overall lack for governmental-sponsored grants directed toward young, independent entrepreneurs and volunteer groups (Young and Grinsfelder, 2011; Legg, 2020; Naude, 2020). Even when financing is obtained, the youth usually has to face additional struggles, including difficulties in obtaining quality certifications for their products. As an example, the United States Food and Drug Administration (FDA) has been overly cautious in approving the use of 3D printed PPE and medical equipment (US Food & Drug Administration, 2020), granting emergency approval to only a handful of organizations (Rodrigo, 2020). Indeed, the majority of FDA COVID-19 actions have focused on relaxing their enforcement guidelines, rather than actively overseeing the development and approving the use of materials and equipment created by youth networks (Flanagan and Ballard, 2020). A similar situation, though to varying degrees, has been observed in other parts of the world, including the European Union (Gierthmuehlen et al., 2020) and the United Kingdom (Emanuel et al., 2020). While aiming to protect the public, these procedures have produced unintended consequences. Specifically, COVID-19 emergency donations and loans provided by multinational organizations such as the United Nations Development Programme, the World Bank and the Interamerican Development Bank often require governments to spend these resources on materials and equipment that are certified by the FDA or the European Union (Ferreira and Mostajo-Radji, 2020). Notable cases are beginning to show the potential of overcoming these sorts of obstacles. For instance, the Bolivian government has worked together with young entrepreneurs to provide local certifications that allows them commercialize 3D printed ventilators in the local market (Los Tiempos Digital, 2020). Similarly, in Colombia, private and university-based ventilators printing projects have gained support from the national medical device regulatory body to begin human trials (Zimmer, 2020). Surmounting governmental regulations currently presents a major impediment to the employment of supplies designed by non-governmental groups and these requirements, in return, hamper governments from supporting initiatives developed by youth networks (Condori, 2020).

## Conclusions

The development of youth networks capable of identifying and undertaking challenges specific to their communities will not only drive forward solutions related directly to their objectives, but consequently advance a common global agenda, independently of direct governmental support (Carosso et al., 2019b). Altogether, the contributions youth networks have made toward addressing aspects of the current pandemic provide a glimpse into the power of non-traditional forms of public health intervention. The examples listed here should serve as models of the benefit of forming such networks, and continued development of youth networks motivated to solve global issues and advance the United Nations SDGs should be kept in focus.

## Author Contributions

All authors listed have made a substantial, direct and intellectual contribution to the work, and approved it for publication.

## Conflict of Interest

MM-R is the Bolivian Science, Technology and Innovation Ambassador. The remaining author declares that the research was conducted in the absence of any commercial or financial relationships that could be construed as a potential conflict of interest.
